# The physiologic complexity of beat‐to‐beat blood pressure is associated with age‐related alterations in blood pressure regulation

**DOI:** 10.1111/acel.13943

**Published:** 2023-08-24

**Authors:** Xin Jiang, Xiaoying Mang, Huiting Zhou, Jingmei Chen, Huiying Tan, Huixia Ren, Baofeng Huang, Lilian Zhong, Lewis A. Lipsitz, Brad Manor, Yi Guo, Junhong Zhou

**Affiliations:** ^1^ Department of Geriatrics Shenzhen People's Hospital Shenzhen China; ^2^ The Second Clinical Medical College Jinan University Shenzhen China; ^3^ The First Affiliated Hospital Southern University of Science and Technology Shenzhen China; ^4^ Hinda and Arthur Marcus Institute for Aging Research Hebrew SeniorLife Boston Massachusetts USA; ^5^ Division of Gerontology Beth Israel Deaconess Medical Center Boston Massachusetts USA; ^6^ Harvard Medical School Boston Massachusetts USA; ^7^ Department of Neurology Shenzhen People's Hospital Shenzhen China; ^8^ Shenzhen Bay Laboratory Shenzhen China

**Keywords:** beat‐to‐beat blood pressure, hypertension, multiscale entropy, orthostatic BP change, physiological complexity

## Abstract

The fluctuations in resting‐state beat‐to‐beat blood pressure (BP) are physiologically complex, and the degree of such BP complexity is believed to reflect the multiscale regulation of this critical physiologic process. Hypertension (HTN), one common age‐related condition, is associated with altered BP regulation and diminished system responsiveness to perturbations such as orthostatic change. We thus aimed to characterize the impact of HTN on resting‐state BP complexity, as well as the relationship between BP complexity and both adaptive capacity and underlying vascular characteristics. We recruited 392 participants (age: 60–91 years), including 144 that were normotensive and 248 with HTN (140 controlled‐ and 108 uncontrolled‐HTN). Participants completed a 10‐min continuous finger BP recording during supine rest, then underwent measures of lying‐to‐standing BP change, arterial stiffness (i.e., brachial‐ankle pulse wave velocity), and endothelial function (i.e., flow‐mediated vasodilation). The complexity of supine beat‐to‐beat systolic (SBP) and diastolic (DBP) BP was quantified using multiscale entropy. Thirty participants with HTN (16 controlled‐HTN and 14 uncontrolled‐HTN) exhibited orthostatic hypotension. SBP and DBP complexity was greatest in normotensive participants, lower in those with controlled‐HTN, and lowest in those in uncontrolled‐HTN (*p* < 0.0005). Lower SBP and DBP complexity correlated with greater lying‐to‐standing decrease in SBP and DBP level (*β* = −0.33 to −0.19, *p* < 0.01), greater arterial stiffness (*β* = −0.35 to −0.18, *p* < 0.01), and worse endothelial function (*β* = 0.17–0.22, *p* < 0.01), both across all participants and within the control‐ and uncontrolled‐HTN groups. These results suggest that in older adults, BP complexity may capture the integrity of multiple interacting physiologic mechanisms that regulate BP and are important to cardiovascular health.

## Graphical abstract

The complex regulation of blood pressure in aging.

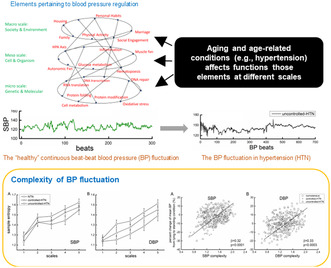

**Abbreviations**
ACEI: angiotensin‐converting enzyme inhibitors
ARB: angiotensin‐II receptor blockers
baPWV: brachial‐ankle pulse wave velocity
BMI: body mass index
BP: blood pressure
CCB: calcium channel blockers
CI: confidence interval
CVD: cardiovascular disease
DBP: diastolic blood pressure
FMD: flow‐mediated dilation
HTN: hypertension
MSE: multiscale entropy
NTN: normotensive
OH: orthostatic hypotension
SBP: supine beat‐to‐beat systolic
SD: standard deviation
TOD: target organ damage

## 1 INTRODUCTION

The maintenance of cardiovascular health is critical to the promotion of healthy aging. The regulation of blood pressure (BP), one of the key functions of the cardiovascular system, is fundamental to the circulation of oxygen, nutrients, and toxic waste in human tissues (Abe et al., 2017; Toth et al., 2017), and is fundamentally dependent on numerous biophysiological processes, including arterial stiffness, endothelial function, baroreflex feedback, and many others. Both aging and hypertension (HTN) are known to impair these processes (Dharmashankar & Widlansky, 2010; Izzo & Taylor, 1999; Sun, 2015; Taddei et al., 1997), leading to hypotensive responses to common daily activities (Lipsitz, 1989). Orthostatic hypotension (OH), as assessed by altered orthostatic change of BP, is one such common consequence of impaired BP regulation (Lipsitz, 1989), contributing to many adverse health‐related consequences including diminished mobility, cognitive impairment, stroke, and falls in older adults (Eigenbrodt et al., 2000; Mol et al., 2019; Ooi et al., 2000; Suemoto et al., 2019).

Traditionally, BP has been characterized by its mean level and/or its variability (i.e., standard deviation [SD]) across multiple days (Qiu et al., 2005; SPRINT MIND Investigators for the SPRINT Research Group et al., 2019). These measurements, while important, do not fully characterize the regulation of BP over multiple temporospatial scales. Indeed, the regulation of human biophysiological processes, including BP, depends upon numerous underlying control elements interacting across multiple scales of time and space (Di Rienzo et al., 2009; Goldberger et al., 2002; O'Connor et al., 2022; Manor & Lipsitz, 2013). BP in particular is continuou·sly regulated and influenced by, for example, the modulation of noncoding RNAs (Marques et al., 2015) at the “micro” scale, baroreflexes (Heusser et al., 2010) at the “meso” scale, and even psychosocial family, and environmental relationships (Wirtz et al., 2006) at the “macro” scale. The resulting dynamics in beat‐to‐beat BP fluctuations, even during rest and over relatively short periods of time, are “complex,” meaning that they contain rich, nonrandom, meaningful information that reflects the interaction of underlying biophysiological control elements acting over multiple temporospatial scales.

The complexity theory of aging proposes that age‐related alterations in the structure and function of a physiologic system reduce the complexity of its output under resting‐state or “basal” conditions, and that diminished resting‐state complexity underscores and thus directly reflects reduced capacity of that system to respond to stressors (Lipsitz & Goldberger, 1992). Multiple signal processing techniques derived from chaos theory (e.g., multiscale entropy [MSE], detrended fluctuation analysis) have been used to characterize the degree of complexity contained within biological series (Costa et al., 2002; Peng et al., 1992). In a series of previous studies, we quantified the complexity of beat‐to‐beat BP fluctuations using MSE, and observed that lower BP complexity correlated with white matter lesions in the brain and the risk of dementia, as well as with frailty and slower walking speeds in older adults (Jiang et al., 2022a, 2022b; Jiang, Cai, et al., 2021; Ma et al., 2021). These observations indicate that BP complexity may be a promising marker of BP regulation and underlying cardiovascular health.

In this study, we aimed to characterize the effects of hypertensive status on BP complexity, and establish the relationships between BP complexity and both the cardiovascular system's capacity to adapt to an orthostatic stressor and underlying vessel function in older adults. We hypothesized that: (1) compared to older normotensive adults, BP complexity would be lower in older adults with controlled HTN, and lowest in those suffering from uncontrolled HTN; and (2) lower BP complexity would correlate with diminished adaptive capacity as measured by a greater orthostatic change in BP from lying to standing, greater arterial stiffness, and worse endothelial function.

## 2 METHODS

### 2.1 Participants

This study was initiated in July 2022 and completed in December 2022. The study team searched potential participants in an electronic medical record database maintained by the Department of Geriatrics, Shenzhen People's Hospital, Shenzhen, China. This repository consisted of information on older adults who completed a clinical visit for routine care between January 1, 2020 and June 30, 2022. The inclusion criteria were as follows: (1) age ≥60 years at the first study visit, and (2) intact mobility as assessed by the ability to walk for at least 30 s without personal/physical assistance. The exclusion criteria were as follows: (1) diagnosis of terminal disease (e.g., cancer) according to their medical record, (2) hospitalization due to any medical procedure (e.g., surgery) within the past 6 months, (3) diagnosis of overt neurological diseases (e.g., dementia, Parkinson's disease, or stroke), (4) chronic kidney disease and dyslipidemia, (5) other cardiovascular diseases (e.g., heart failure, coronary artery disease), or (6) an inability to understand the study protocol. All experimental protocols were approved by the Institutional Review Board of Shenzhen People's Hospital and carried out in accordance with the guidelines of the Declaration of Helsinki. All participants provided written consent prior to participating in study procedures.

### 2.2 Study protocol

Older adults from the electronic medical record database who were willing to participate in this study completed one screening visit, and if eligible, two study visits in person at the Department of Gerontology within the hospital. These three visits were each separated by at least 24 h. On the screening visit, one research assistant first introduced the protocol of this study. Participants then completed questionnaires to provide their most recent clinical information that was not included in their previous medical record, as well as their understanding of this study, to determine their eligibility. On the first study visit, eligible participants then completed a series of questionnaires to assess demographics (e.g., age, sex, body mass index [BMI]), health habits (e.g., use of alcohol, i.e., number of drinks taken per week [Morse & Flavin, 1992] and smoking [Freedman et al., 2011]), complaints of dizziness with postural change (i.e., yes or no), and the duration of HTN. One research team member completed these procedures on this visit. On the second study visit, participants completed a beat‐to‐beat finger BP assessment, a lying‐to‐standing test of OH, and measurements of arterial stiffness and endothelial function under the administration of two research team members to ensure the quality of the assessment and the safety of participants. These tests were completed on the morning before taking any antihypertensive medications. Throughout the study, participants were asked to refrain from eating or drinking caffeinated beverages 24 h before and throughout this study.

#### 2.2.1 Hypertensive characteristics

The hypertensive status of each participant was assessed from the clinical records and confirmed on the screening visit. Specifically, HTN was characterized as systolic BP (SBP) ≥140 mmHg and/or diastolic BP (DBP) ≥90 mmHg at the brachial artery of the left arm using an automated sphygmomanometer. We thus categorized participants into normotensive (NTN), controlled‐hypertensive (controlled‐HTN) (i.e., those who were diagnosed with HTN and actively taking related medication, yet whose SBP and DBP values were below the thresholds for HTN), and uncontrolled‐hypertensive (uncontrolled‐HTN) (i.e., those who met the BP thresholds for hypertensive whether or not they were using antiHTN medication). We also categorized antiHTN medication into calcium channel blockers, angiotensin‐converting enzyme inhibitors, angiotensin‐II receptor blockers, beta‐blockers, and diuretics. The number of antihypertensive medications each participant used simultaneously, and the duration of HTN diagnosis were recorded and included as covariates in the following analyses.

#### 2.2.2 BP recordings

On the morning of the second visit, each participant first completed the assessment of “resting state” beat‐to‐beat BP fluctuations in a quiet assessment room with two study members. During the recording of BP, participants were instructed to avoid talking and refrain from moving as much as possible. Items that may interfere with the recording, such as mobile phone, were stored securely outside the room. Continuous beat‐to‐beat SBP and DBP time series were recorded using Finometer PRO system (Finapres Medical Systems B.V.) secured to the middle finger of the left hand with the participant lying supine. The series was recorded for 10–15 min to ensure enough data points (as the number of beats per minute may vary across individuals). The sampling frequency was 100 Hz (Guelen et al., 2008). The BeatScope software package (Finapres Medical Systems B.V.) was then used to obtain the SBP and DBP values of each beat. All BP recordings consisted of at least 700 continuous beats. For the preprocessing of BP time series, we followed the procedure that has been used in our previous studies (Jiang, Cai, et al., 2021; Jiang, Guo, et al., 2021; Ma et al., 2021). Specifically, outliers were identified as BP values greater or lower than the series mean ± two times the SD. These outliers were then interpolated using the mean of the series after the removal of them (Dauphinot et al., 2013; Douma & Gumz, 2018). The BP complexity and variability were then calculated as described below using these preprocessed BP time series of 700 sampling points.

#### 2.2.3 Assessment of orthostatic BP change and hypotension

The OH status of participants was unknown before this study. No participants were being treated for OH at the time of the study. After the continuous beat‐to‐beat BP recording, participants completed the lying‐to‐standing assessment of OH in the same room following an established protocol (Freeman et al., 2012). Specifically, participants rested in a lying position for 10 min, and then actively moved to a standing position as quickly as possible. They were then asked to remain motionless for 3 min. The level of their SBP and DBP was measured using a BP monitor (OMRON Healthcare, Inc.) when lying and in standing conditions once each minute. If a maximum reduction of SBP of at least 20 mmHg and/or DBP of 10 mmHg was observed within 3 min of standing as compared to the average BP level in the lying condition, the participant was identified as having OH (Freedman et al., 2011). The percent change of BP level from lying to standing position was also calculated and used as the outcome measuring the orthostatic change of BP regulation.

#### 2.2.4 Assessment of arterial stiffness and vascular endothelial function

Arterial stiffness was assessed by measuring the left‐ and right‐side brachial‐ankle pulse wave velocity (baPWV) (Omron). The average baPWV was then obtained by averaging left and right baPWV (Turin et al., 2010; Yamashina et al., 2002). Vascular endothelial function was assessed using the flow‐mediated dilation (FMD) (Unex) when participants were in a supine resting state (Thijssen et al., 2011). Both average baPWV and FMD were used in the following analyses.

#### 2.2.5 BP complexity

The complexity of SBP and DBP series was quantified using multiscale entropy (MSE), a widely used technique that quantifies the entropy in biophysiological time series over multiple temporal scales. Specifically, the preprocessed beat‐to‐beat SBP and DBP time series was first “coarse‐grained” from Scale 1 to 5 by dividing the original series into nonoverlapping windows of length equaling a scale factor from 1 to 5 sampling points (Costa et al., 2005). For example, the series at Scale 1 was the raw series consisting of 700 points; Scale 2 used the average of every two measures and consisted of 350 points; and so on to Scale 5, which was constructed by averaging every five nonoverlapping points, thereby creating a new series of 140 points (i.e., 700 points/5). The sample entropies of each “coarse‐grained” series were then calculated, as defined by the negative of the natural logarithm of the conditional probability that a series, having repeated itself for *m* consecutive data points (*m* is the length of the defined pattern; and the repeatability is determined by the number of patterns in this series with a difference from the defined patterns smaller than the SD of the series multiplied by a tolerance parameter, *r*), will also repeat itself for *m + 1* points within the same tolerance without self‐matches (Costa et al., 2002, 2005; Zhou et al., 2017). Following the same procedure of MSE calculation in the previous studies (Costa et al., 2005; Jiang, Cai, et al., 2021; Jiang, Guo, et al., 2021; Zhou et al., 2017), we computed the sample entropy of each coarse‐grained series by choosing the parameter of tolerance *r* = 0.15 and number of matching points *m* = 2. Notably, to obtain reliable estimation of entropy, Costa et al. (2002) recommended that the number of data points in the coarse‐grained time series at the largest scale should be at least 10^m^ to 17^m^ (here *m* = 2, so *n* = 100–289). On Scale 5, the number of data points was 140, greater than the recommended number, suggesting the estimation of the entropy was reliable.

**FIGURE 1:**
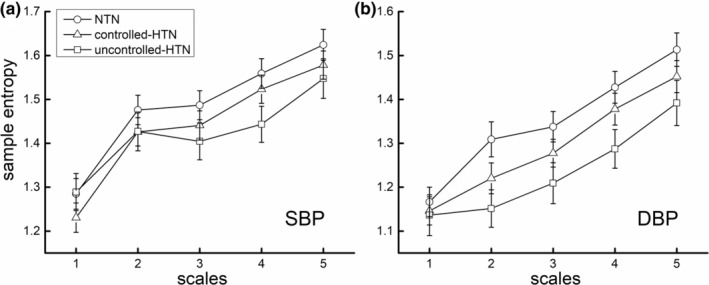
The multiscale entropy curves (mean and standard error) of supine beat‐to‐beat systolic (SBP) (a) and diastolic blood pressure (DBP) (b) of each group of hypertensive status (i.e., normotensive [NTN], controlled‐hypertensive [controlled‐HTN], and uncontrolled‐HTN). Entropy at Scale 1 (i.e., traditional sample entropy) was relatively similar between groups, yet appeared to differ by group at larger scales.

Figure 1 shows the MSE curves of each group. By observing the pattern of these curves and following that was used in previous studies (Costa et al., 2005; Hadoush et al., 2019), the BP complexity was defined as the averaged entropy across five scales, with lower values reflective of lower complexity.

The variability of each SBP and DBP series was also calculated as the SD about the mean value and used in the following analyses.

### 2.3 Statistical analysis

Statistical analyses were performed with JMP 16 software (SAS Institute). The significant level of the analyses was set at *p* < 0.05.

We first categorized participants into three groups, that is, NTN, controlled‐HTN, and uncontrolled‐HTN. The normality of the data was examined using the Shapiro–Wilk test, and the homogeneity of variance was examined using the Levene's test. To compare the demographic and clinical characteristics between groups, we used one‐way ANOVA models when data were normally distributed and the Kruskal–Wallis test when the data were not normally distributed. The Tukey's post hoc analysis was used to compare the factor means of significant models. For categorical variables (e.g., sex), we used chi‐squared tests.

To test the hypothesis that the presence of HTN would be associated with lower BP complexity, we used one‐way ANOVA models when the data were normally distributed. The model factor was group (i.e., NTN, controlled‐HTN, and uncontrolled‐HTN) and the dependent variable was SBP and DBP complexity (examined in separate models). All models were adjusted for age, sex, BMI, alcohol use (i.e., men who took more than 14 drinks per week or women took more than seven drinks per week), smoking status (i.e., smoker or nonsmoker), and mean BP level (SBP and DBP, respectively), which were all believed to contribute to the regulation of BP. Tukey's post hoc analysis was used to compare the factor means of significant models. When data were not normally distributed, we used the Kruskal–Wallis test. Secondarily, similar models were used to examine the relationship between hypertensive status and the mean and variability of SBP and DBP, arterial stiffness (i.e., average baPWV), and vascular endothelial function (i.e., FMD).

To test the hypothesis that BP complexity would be associated with the orthostatic change in BP as measured using the percent change of BP level from lying to standing, we performed primary linear regression analyses for SBP and DBP complexity in separate models across all participants. Age, sex, BMI, group, alcohol use, smoking status, and mean BP level (SBP and DBP, respectively) were included as the covariates. Secondarily, we performed similar analyses separately for each group. Uniquely, within the controlled‐HTN and uncontrolled‐HTN groups, we also included the number of prescribed antiHTN medications and the duration of HTN diagnosis as additional covariates. The linear regression analyses were also performed to examine the relationship of the variability and mean level of SBP and DBP to their complexity and the percent change of their level from lying to standing condition, respectively. Age, sex, BMI, group, alcohol use, and smoking status were included as the covariates.

To test the hypothesis that BP complexity would be associated with arterial stiffness (as assessed using average baPWV) and endothelial function (as assessed using FMD), we primarily performed similar linear regression analyses as described above. Age, sex, BMI, group, alcohol use, smoking status, and mean BP level (SBP and DBP, respectively) were included as the covariates. We also explored such relationships within each group of hypertensive status. Within the controlled‐HTN and uncontrolled‐HTN groups, we included the number of prescribed antiHTN medications and duration of HTN diagnosis as additional covariates. Similar analyses were also performed to examine the relationship of the variability and mean level of SBP and DBP to average baPWV and FMD.

## 3 RESULTS

A total of 392 eligible participants completed the assessments. All acquired data were included in the analyses. One hundred and forty‐four participants were NTN, and 248 were hypertensive. Of these, 140 were defined as controlled‐HTN and 108 uncontrolled‐HTN. Table 1 shows the demographic, clinical, and functional characteristics of the entire cohort and within each subgroup. Shapiro–Wilk tests indicated that all variables were normally distributed (*p* > 0.45). One‐way ANOVA models revealed that age (*p* = 0.0007) and BMI (*p* = 0.0006) were significantly different between groups. Post hoc analyses revealed that compared to the NTN group, participants in controlled‐ and uncontrolled‐HTN groups were older and had greater BMI. The number of women, education, and the use of alcohol and smoking were similar between groups (*p* = 0.09–0.48). Based upon the results of lying‐to‐standing test, no participants in the NTN group had OH. However, 16 (11.4%) and 14 (12.9%) participants demonstrated OH in the controlled‐ and uncontrolled‐HTN group, respectively. The prevalence of OH across these two groups was similar (*p* = 0.51). The portion of participants who reported experiencing dizziness in daily life was relatively greater in uncontrolled‐HTN (8.3%) group as compared to the other two (1.4%–2.9%).

**TABLE 1:** Demographic and clinical characteristics and the outcomes of vascular function in this population.

*n* (%) or mean ± SD	Total (*n* = 392)	Normotensive (*n* = 144)	HTN (*n* = 218)		*p*‐Value
			Controlled‐HTN (*n* = 140)	Uncontrolled‐HTN (*n* = 108)	
Age (years)	71.5 ± 7.4	69.2 ± 6.8^A^	71.1 ± 7.5^B^	72.5 ± 7.4^B^	**0.0007**
Sex					
Female	214 (54.5)	76 (52.8)	75 (53.6)	63 (58.3)	0.59
Male	178 (44.0)	68 (42.2)	65 (46.4)	45 (41.7)	
Education (years)	9.6 ± 4.7	10.0 ± 4.5	9.4 ± 4.9	8.6 ± 5.1	0.09
BMI	24.1 ± 3.3	23.5 ± 3.3^A^	25.2 ± 3.6^B^	25.2 ± 3.8^B^	**0.0006**
Smoking (*n* = smokers)	51 (13.0)	23 (16.0)	15 (10.7)	13 (12.0)	0.34
Alcohol (*n* = alcoholic)	36 (9.2)	12 (8.3)	16 (11.4)	8 (7.4)	0.48
Number of OH	30 (0)	0 (0)	16 (11.4)	14 (12.9)	0.51
SBP complexity	1.42 ± 0.26	1.51 ± 0.26^A^	1.44 ± 0.30^B^	1.36 ± 0.27^C^	**0.0004**
DBP complexity	1.34 ± 0.25	1.41 ± 0.28^A^	1.33 ± 0.29^B^	1.23 ± 0.29^C^	**<0.0001**
Mean SBP (mmHg)	133.8 ± 12.3	126.6 ± 10.3^A^	126.9 ± 9.7^A^	152.5 ± 9.9^B^	**<0.0001**
Mean DBP (mmHg)	77.2 ± 7.8	75.4 ± 6.2^A^	74.9 ± 7.9^A^	83.2 ± 9.5^B^	**<0.0001**
SBP variability	5.17 ± 1.46	5.14 ± 0.14	5.23 ± 0.13	5.24 ± 0.30	0.33
DBP variability	2.99 ± 1.03	3.02 ± 0.55	3.03 ± 0.45	2.98 ± 0.67	0.47
Average baPWV (m/s)	17.3 ± 3.2	16.4 ± 3.3^A^	17.6 ± 3.2^B^	20.4 ± 4.4^C^	**<0.0001**
FMD (%)	3.4 ± 2.3	4.1 ± 2.1^A^	3.4 ± 1.9^B^	3.0 ± 1.9^B^	**0.004**
BP change (%)					
SBP	1.7 ± 7.9	0.1 ± 6.9	1.0 ± 6.9	1.3 ± 7.2	0.68
DBP	0.2 ± 8.7	−0.9 ± 7.5	−1.4 ± 7.7	0.2 ± 7.0	0.21
Self‐report of dizziness	15 (3.8)	2 (1.4)	4 (2.9)	9 (8.3)	0.12
Hypertension history (years)	n.a	n.a.	12.5 ± 8.6	11.5 ± 8.5	n.a.
Antihypertensive medication (*n*)					
CCB	n.a.	n.a.	89 (61.8)	73 (67.6)	n.a.
ACEI	n.a.	n.a.	7 (5.0)	4 (3.7)	n.a.
ARB	n.a.	n.a.	42 (30.0)	35 (32.4)	n.a.
BB	n.a.	n.a.	40 (28.6)	28 (25.9)	n.a.
Diuretics	n.a.	n.a.	9 (6.4)	9 (8.3)	n.a.
Number of medication (*n*)					
1	n.a.	n.a.	70 (50.0)	57 (52.8)	n.a.
2	n.a.	n.a.	53 (37.9)	35 (32.4)	n.a.
3	n.a.	n.a.	14 (10.0)	14 (13.0)	n.a.
4	n.a.	n.a.	3 (2.1)	2 (1.8)	n.a.
5	n.a.	n.a.	0 (0)	0 (0)	n.a.

Abbreviations: ACEI, angiotensin‐converting enzyme inhibitors; ARB, angiotensin‐II receptor blockers; baPWV, brachial‐ankle pulse wave velocity; BB, beta‐blockers; BMI, body mass index; CCB, calcium channel blockers; DBP, diastolic blood pressure; FMD, flow‐mediated dilation; HTN, hypertensive; OH, orthostatic hypotension; SBP, systolic blood pressure.

*Note*: BP change (%): the percent change of blood pressure from lying to standing condition in lying‐to‐standing test; Negative value reflected a decrease of blood pressure from lying to standing condition. A, B, C: different letters reflected where the significant difference was based upon the results of post hoc Tukey's test.

Linear regression analyses revealed that neither SBP nor DBP complexity was significantly associated with mean SBP or DBP level (*β* = −0.01 to 0.08, *p* = 0.31–0.48, 95% confidence interval [CI]: −0.08 to 0.14), or the variability of SBP and DBP (*β* = 0.04–0.07, *p* = 0.28–0.87, 95% CI: 0.01–0.13) across all the participants or within each group of hypertensive status.

### 3.1 The association between hypertensive status and BP complexity, lying‐to‐standing task performance and vessel function

Figure 1 depicts the average (±standard error) MSE curves for each group. It can be observed that the sample entropy is relatively similar between groups at Scale 1, but noticeably different across groups starting from Scale 2. Specifically, the sample entropy was highest in the NTN group, lower in the controlled‐HTN group, and lowest in the uncontrolled‐HTN group.

**FIGURE 2:**
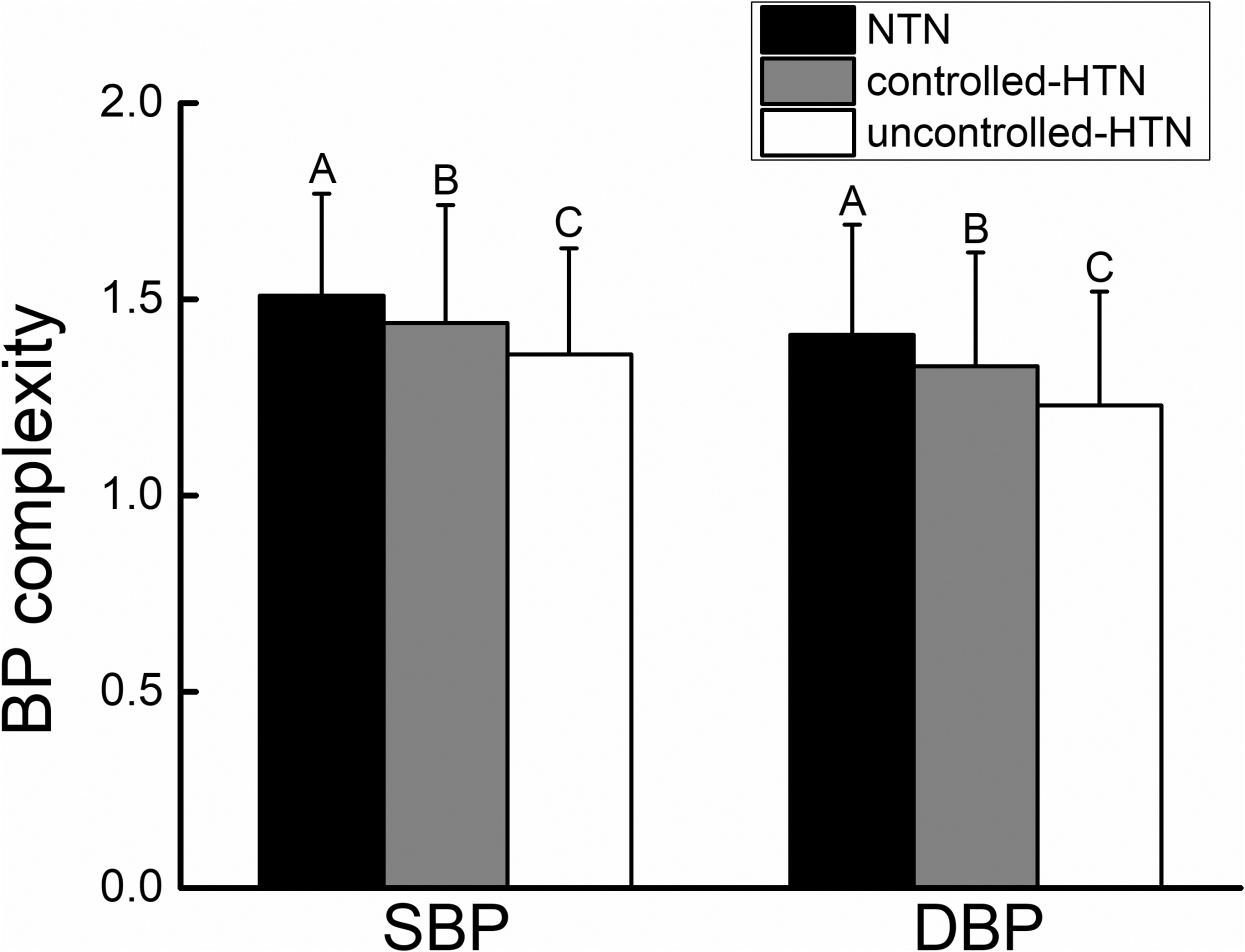
The effects of hypertensive status on the supine beat‐to‐beat systolic (SBP) and diastolic blood pressure (DBP) complexity. Compared to normotensive (NTN) group, the SBP (*p* = 0.0004) and DBP (*p* < 0.0001) complexity were significantly lower in the controlled‐hypertensive (controlled‐HTN) group, and were still lower in the uncontrolled‐HTN group. Such effects were independent from the influence of age, sex, BMI, alcohol and smoking usage, number of antihypertension (HTN) medication used simultaneously, and duration of HTN diagnosis. Different letters (i.e., A, B, and C) on the figure revealed where the significant difference was based upon the results of post hoc Tukey's test.

The ANOVA models indicated significant group effects on both SBP (*p* = 0.0004) and DBP (*p* < 0.0001) complexity (Figure 2). No significant influences of covariates, including age, sex, BMI, alcohol use, smoking status, and mean BP level, on BP complexity were observed (*p* = 0.28–0.89). Post hoc analyses revealed that compared to the NTN group, both SBP and DBP complexity were significantly lower in the controlled‐HTN group, and lowest in the uncontrolled‐HTN group (Table 1).

Secondary analyses indicated that compared to the NTN and controlled‐HTN group, the mean levels of SBP and DBP were significantly greater in the uncontrolled‐HTN group (*p* < 0.0001); however, no such difference was observed for the variability of SBP (*p* = 0.33) and DBP (*p* = 0.47).

Across all the participants, the percent change of SBP level from lying to standing condition was 1.7% ± 7.9% and that of DBP was 0.2% ± 8.7%. This percent change of SBP (*p* = 0.68) and DBP (*p* = 0.21) did not significantly differ between groups (Table 1). On the other hand, group differences were observed in average baPWV (*p* < 0.0001) and FMD (*p* = 0.004). Compared to the NTN group, the average baPWV was greater in the controlled‐HTN and greatest in the uncontrolled‐HTN group. FMD was lower in both HTN groups as compared to the NTN group (Table 1).

### 3.2 The association between BP complexity and the percent change of BP level in the lying‐to‐standing test

**FIGURE 3:**
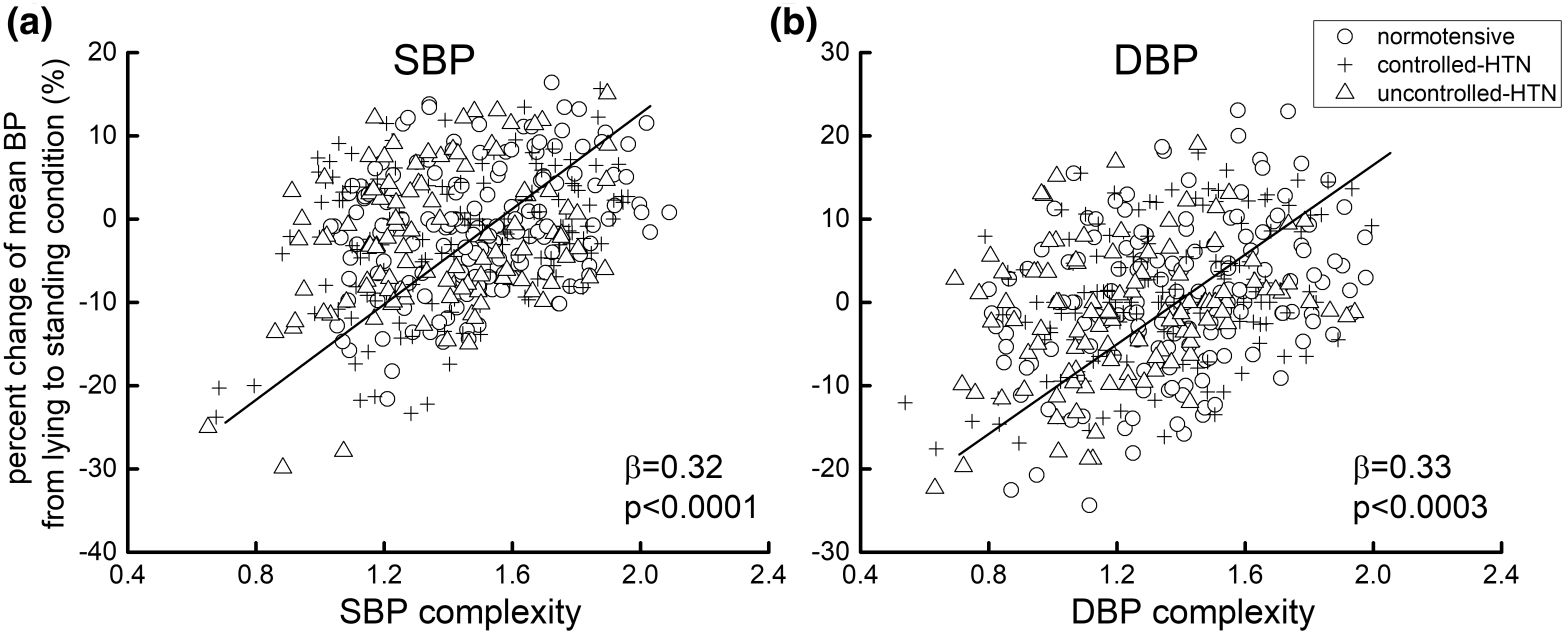
The association between BP complexity and percent change of BP level from lying to standing condition. The linear regression models showed that across all groups, participants with lower supine beat‐to‐beat systolic (SBP) (*β* = −0.32, *p* < 0.0001) (a) and diastolic blood pressure (DBP) (*β* = −0.33, *p* = 0.0003) (b) complexity had greater percent reduction of SBP and DBP levels from lying to standing condition in lying‐to‐standing test respectively.

Linear regression analyses revealed significant associations between BP complexity and the percent change of SBP and DBP level experienced during the lying‐to‐standing test (SBP: *β* = −0.32, 95% CI: −0.40 to −0.22, *p* < 0.0001; DBP: *β* = −0.33, 95% CI: −0.41 to −0.23, *p* = 0.0003). Across all participants, individuals with lower SBP or DBP complexity exhibited greater percent reduction of SBP or DBP level from the lying to the standing condition, respectively (Figure 3). This relationship was not significantly different between groups (SBP: *p* = 0.24; DBP: *p* = 0.49) and no significant influences of age, sex, BMI, alcohol use, smoking status, and mean BP level (*p* = 0.18–0.45). Secondary analyses further indicated that the relationship between BP complexity and the orthostatic change of BP was also significant within each group when analyzed separately (Table 2).

**TABLE 2:** The association between BP complexity, orthostatic change of BP, baPWV, and FMD within each group.

	NTN	Controlled HTN	Uncontrolled‐HTN
Orthostatic change of BP			
SBP	*β* = −0.28, *p* = 0.007	*β* = −0.23, *p* = 0.005	*β* = −0.22, *p* = 0.01
DBP	*β* = −0.24, *p* = 0.003	*β* = −0.26, *p* = 0.004	*β* = −0.19, *p* = 0.01
BaPWV			
SBP	*β* = −0.29, *p* = 0.005	*β* = −0.25, *p* = 0.01	*β* = −0.18, *p* = 0.009
DBP	*β* = −0.26, *p* = 0.0004	*β* = −0.23, *p* = 0.008	*β* = −0.22, *p* = 0.007
FMD			
SBP	*β* = 0.17, *p* = 0.01	*β* = 0.24, *p* = 0.01	*β* = 0.17, *p* = 0.009
DBP	*β* = 0.20, *p* = 0.004	*β* = 0.22, *p* = 0.01	*β* = 0.19, *p* = 0.01

Abbreviations: baPWV, brachial‐ankle pulse wave velocity; DBP, diastolic blood pressure; FMD, flow‐mediated dilation; HTN, hypertensive; NTN, normotensive; SBP, systolic blood pressure.

Across all participants, as well as within each group, neither mean BP level (SBP: *β* = 0.06–0.1, 95% CI: −0.06 to 0.13, *p* = 0.15–0.39; DBP: *β* = 0.08–0.11, 95% CI: −0.05 to 0.15, *p* = 0.11–0.38) nor the BP variability (SBP: *β* = −0.05 to −0.07, 95% CI: −0.15 to 0.05, *p* = 0.27–0.45; DBP: *β* = −0.04 to 0.06, 95% CI: −0.14 to 0.06, *p* = 0.76–0.89) were significantly associated with the percent change of SBP and DBP levels induced by the lying‐to‐standing test, respectively.

### 3.3 The association between BP complexity and vessel characteristics

**FIGURE 4:**
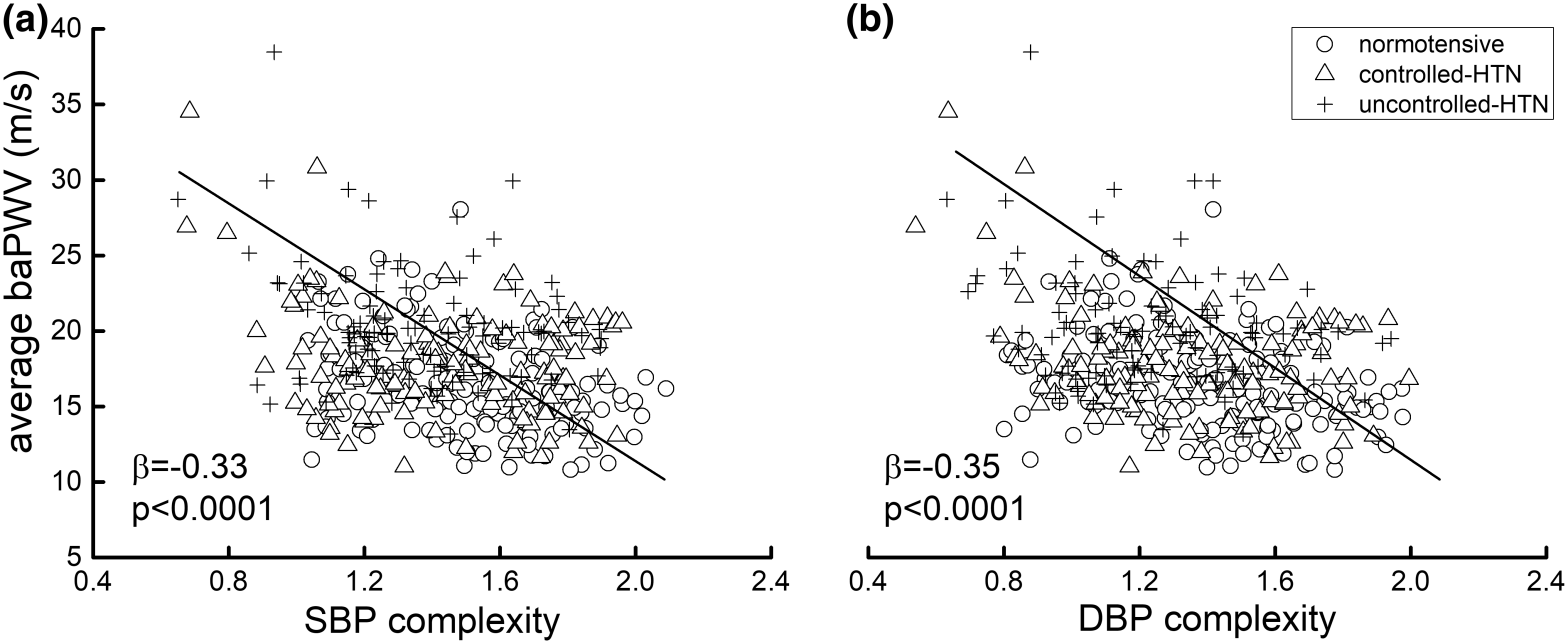
The association between BP complexity and arterial stiffness. The linear regression models showed that across all groups, participants with lower supine beat‐to‐beat systolic (*β* = −0.33, *p* < 0.0001) (a) and diastolic blood pressure (*β* = −0.35, *p* = 0.0003) (b) complexity had greater arterial stiffness as assessed by higher average brachial‐ankle pulse wave velocity (baPWV).

**FIGURE 5:**
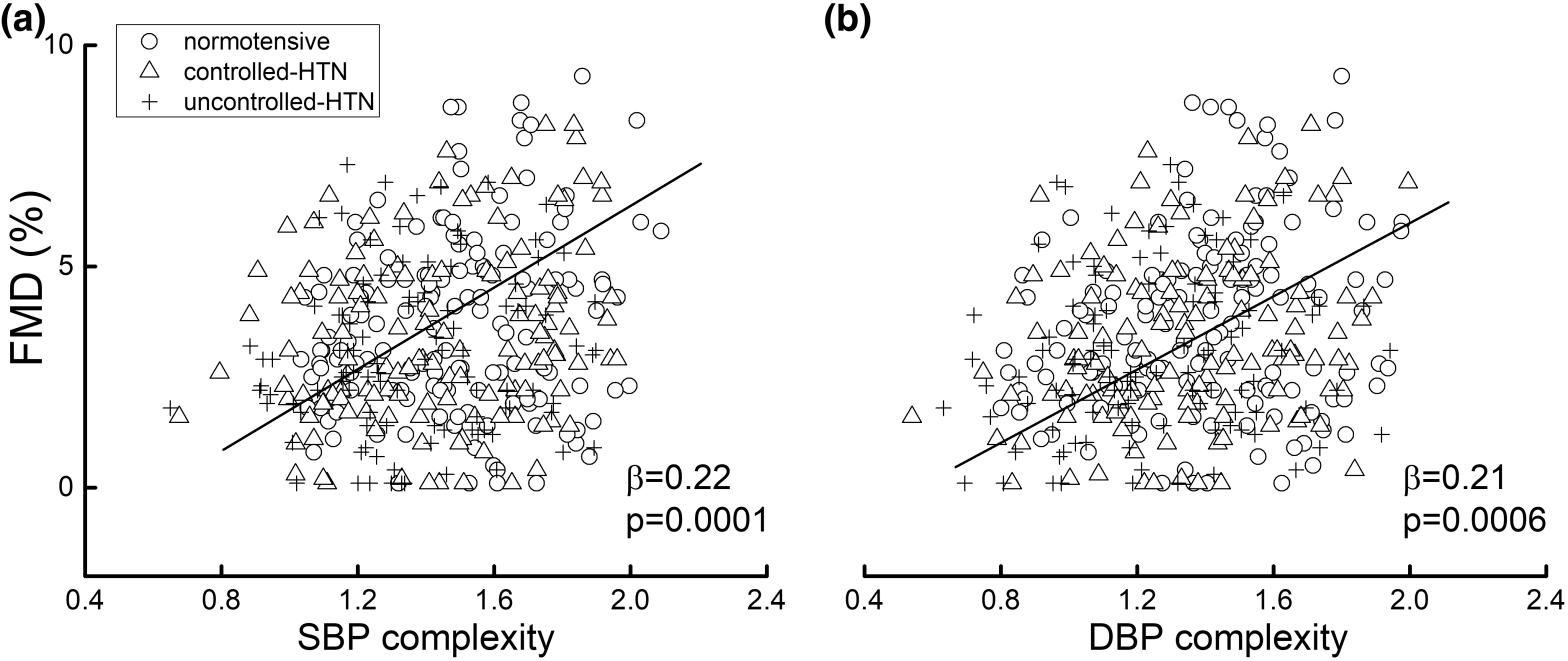
The association between BP complexity and endothelial function. The linear regression models showed that across all groups, participants with lower supine beat‐to‐beat systolic (*β* = 0.22, *p* = 0.0001) (a) and diastolic blood pressure (*β* = 0.21, *p* = 0.0006) (b) complexity had poorer endothelial function as assessed by higher flow‐mediated dilation (FMD).

Adjusted linear regression analyses demonstrated a significant association between BP complexity and average baPWV (SBP: *β* = −0.33, 95% CI: −0.39 to −0.24, *p* < 0.0001; DBP: *β* = −0.35, 95% CI: −0.41 to −0.23, *p* < 0.0001). This relationship did not significantly differ between groups (SBP: *p* = 0.35; DBP: *p* = 0.55). Across all participants, individuals with lower SBP or DBP complexity had greater baPWV (i.e., higher arterial stiffness) (Figure 4). Similar linear regression models demonstrated a significant association between the BP complexity and FMD (SBP: *β* = 0.22; 95% CI: 0.11–0.27, *p* = 0.0001; DBP: *β* = 0.21, 95% CI: 0.10–0.25, *p* = 0.0004; Figure 5); that is, both lower SBP and DBP complexity were associated with lower FMD (i.e., poorer vascular endothelial function). Secondary analyses further revealed that this relationship held within each group when analyzed separately (Table 2). Finally, linear regression models revealed that across all the participants, as well as within each group, neither BP variability (SBP: *β* = −0.04 to 0.08, 95% CI: −0.11 to 0.12, *p* = 0.37–0.55; DBP: *β* = −0.06 to 0.06, 95% CI: −0.13 to 0.10, *p* = 0.77–0.87), nor the mean BP (SBP: *β* = −0.06 to 0.1, 95% CI: −0.09 to 0.14, *p* = 0.18–0.49; DBP: *β* = −0.07 to 0.10, 95% CI: −0.08 to 0.14, *p* = 0.16–0.67) were significantly associated with the baPWV or FMD, respectively.

## 4 DISCUSSION

This study provided evidence that the physiologic complexity of resting‐state BP fluctuations was closely associated with hypertensive status in older adults, greater orthostatic change in BP from lying to standing, increased arterial stiffness, and impaired vascular endothelial function. These observations provide novel insights into the regulation of BP and suggest that BP complexity may capture the influences of HTN on this important physiological procedure in humans.

The results of this study revealed that older adults with controlled‐ or uncontrolled‐HTN presented with significantly lower BP complexity as compared to NTN. Moreover, whether examined across the entire cohort or within each HTN status group separately, participants with lower BP complexity during rest exhibited worse vessel function and diminished adaptive capacity as defined by a greater reduction in BP induced by the lying‐to‐standing test. A central premise of the “complexity theory of aging” is that in older adults, reduced complexity in system output under resting or basal conditions is directly related to diminished capacity of that system to respond and adapt to stressors (Lipsitz & Goldberger, 1992). For BP regulation, during and immediately after a change in posture, the return of venous blood flow to the heart is decreased, and ventricular filling is reduced. In healthy individuals, the reduction of blood volume and arterial pressure is detected by cardiopulmonary receptors and baroreceptors, initiating a baroreflex‐mediated compensatory sympathetic activation, and decreased parasympathetic activation. This autonomic compensatory mechanism thus increases venous return and vascular resistance in order to rapidly stabilize BP (Magkas et al., 2019). In older adults with HTN, this compensatory action may be disrupted due to autonomic nervous dysfunction, blood volume depletion, and/or systemic vasodilation pertaining to HTN, resulting in an increased incidence of OH (Biaggioni, 2018) that may cause severe symptoms including dizziness and lightheadedness (Magkas et al., 2019). Intriguingly, no such relationships were observed between traditional BP measures (i.e., mean BP level and BP variability) and vessel health or adaptive capacity. Taken together, these observations thus indicate that compared to traditional “single‐scale” metrics, the degree of BP complexity contained with resting‐state BP fluctuations may be particularly sensitive to multiple aspects of cardiovascular system health in older adults. Future work is needed to explore age‐related biophysiological alteration that underneath decreased BP complexity and how these alterations my associate with and/or lead to diminished adaptive capacity.

Increased arterial stiffness and endothelial dysfunction in older adult population have been linked to increased risk of cardiovascular conditions (e.g., cardiovascular disease [CVD], heart failure) (Gilani et al., 2020; Ohkuma et al., 2017; Widlansky et al., 2003; Yamashina et al., 2003). Ohkuma et al. (2017), for example, observed that in older adults without CVD, compared to lowest quintile of baPWV, those with baPWV in the highest quintile at baseline were 3.5 times more likely to develop CVD in the following 6 years. The results of this study that lower BP complexity is associated with greater arterial stiffness (i.e., higher baPWV) and worse endothelial function (i.e., lower FMD) across all the groups indicate that BP complexity may be an early indicator to the risk of age‐related cardiovascular conditions. Longitudinal studies are therefore warranted to determine whether baseline BP complexity, or changes in this measure over time, can predict the likelihood of developing CVD and suffering related adverse health outcomes in the future.

The observations of this cross‐sectional study, that BP complexity was closely associated with cardiovascular health in older adults, suggest that BP complexity may be a suitable target for the management of BP within this particularly vulnerable population. Compared to the normotensive group, the controlled‐HTN group still exhibited significantly lower BP complexity. Moreover, the proportion of controlled‐HTN participants with OH was similar to that of the uncontrolled‐HTN group. This indicates that although the use of antiHTN medication may reduce BP, it does not necessarily influence the multiscale control of BP and the related capacity to detect, respond, and adapt to stressors over time. In other words, in addition to lowering average BP level and/or reducing the degree of BP variability, restoring, or increasing BP complexity may further enhance BP regulation and cardiovascular function. Numerous studies have shown that the age‐related loss of physiological complexity is not obligatory but can be restored by appropriate interventions and thereby improve cardiovascular function (Millar et al., 2013; Zhou et al., 2016, 2020). It is thus worthwhile to examine if the medications that are reported to manage both HTN and OH (Juraschek & Biaggioni, 2022; Kaufmann et al., 2014) modulate BP complexity, and if such changes in BP complexity are associated with changes in OH, adaptive capacity, and other HTN‐related outcomes.

This study consisted of participants without any other CVD or target organ damage (TOD) (e.g., microalbuminuria). Future studies are thus warranted to explore the potential impacts of CVD and TOD on BP complexity. Though it has been demonstrated that complexity metrics often capture the consequences of biological aging on the biophysiological procedures of humans (Manor & Lipsitz, 2013), this study was cross‐sectional in nature and does not provide direct evidence to this effect. Future studies with longitudinal designs are thus warranted. Moreover, in this study, we only measured the change of BP level from lying to standing to examine adaptive capacity. Other outcomes from the lying‐to‐standing test may also be worth for further study. For example, the time to recover to normal BP level has been used to characterize subtypes of OH (e.g., delayed OH; i.e., a BP drop after 3 min after standing upright [Gibbons & Freeman, 2015]). Additionally, recent research efforts have shown that orthostatic HTN (Kario, 2013), a condition in which the change of BP is in an “inversed” direction compared to OH (i.e., increase of BP from lying to standing), is also a cardiovascular risk factor. It is thus of great importance to characterize the physiologic complexity of BP regulation more explicitly in different vascular conditions (e.g., subtypes of OH), and its relationships to the capacity of vascular system to adapt to perturbations in these conditions. Finally, it is important to note that we calculated the averaged entropy across just five scales to quantify complexity. Future work is thus needed to determine the comparative sensitivity of this approach, relative to other approaches such as quantification of the slope of the MSE curve (Costa et al., 2005). New approaches to the estimation of entropy from short‐length time series (e.g., distribution entropy [Li et al., 2015], fuzzy entropy [Azami et al., 2019]) should also be explored. Nevertheless, the results of this work suggest that BP complexity holds great promise to serve as a marker for cardiovascular health within older adults.

## AUTHOR CONTRIBUTIONS

*Conception and design*: Junhong Zhou, Yi Guo, and Xin Jiang. *Acquisition of data*: Xiaoying Mang, Huiting Zhou, Jingmei Chen, Huiying Tan, Huixia Ren, Baofeng Huang, Lilian Zhong, and Xin Jiang. *Analysis and interpretation of data*: Junhong Zhou, Brad Manor, Lewis A. Lipsitz, Yi Guo, and Xin Jiang. *Drafting and revising the article*: All authors.

## CONFLICT OF INTEREST STATEMENT

The authors declare no conflict of interest.

## Data Availability

The data that support the findings of this study are available from the Department of Geriatrics, Shenzhen People's Hospital, Shenzhen, Guangdong, China. All de‐identified raw data of each participant in this study are accessible upon reasonable request following appropriate research ethical approvals and with permission of authors.
